# Wnt6 is required for maxillary palp formation in *Drosophila*

**DOI:** 10.1186/1741-7007-11-104

**Published:** 2013-10-03

**Authors:** Nikolaos Doumpas, Gáspár Jékely, Aurelio A Teleman

**Affiliations:** 1 German Cancer Research Center (DKFZ), Heidelberg, Germany; 2 Max Planck Institute for Developmental Biology, Spemannstrasse 35, 72076 Tuebingen, Germany

**Keywords:** *Drosophila*, Wnt6, Maxillary palps

## Abstract

**Background:**

Wnt6 is an evolutionarily ancient member of the Wnt family. In *Drosophila*, Wnt6 loss-of-function animals have not yet been reported, hence information about fly Wnt6 function is lacking. In wing discs, Wnt6 is expressed at the dorsal/ventral boundary in a pattern similar to that of wingless, an important regulator of wing size. To test whether Wnt6 also contributes towards wing size regulation, we generated Wnt6 knockout flies.

**Results:**

Wnt6 knockout flies are viable and have no obvious defect in wing size or planar cell polarity. Surprisingly, Wnt6 knockouts lack maxillary palps. Interestingly, Wnt6 is absent from the genome of hemipterans, correlating with the absence of maxillary palps in these insects.

**Conclusions:**

Wnt6 is important for maxillary palp development in *Drosophila*, and phylogenetic analysis indicates that loss of Wnt6 may also have led to loss of maxillary palps on an evolutionary time scale.

## Background

During animal development, tissue growth is tightly controlled, leading to adults of defined sizes and proportions. Tissue growth is regulated by a combination of patterning cues, which give each tissue a specific identity and hence size, and environmental cues, which are sensed through nutrient responsive pathways and act to proportionately scale the whole animal [1-4]. Despite intense interest in understanding how tissue growth is controlled, the underlying molecular mechanisms are only partly understood. The *Drosophila* wing has become one model system that is frequently used to study this problem. Growth of the fly wing is promoted via signals emanating from the anterior/posterior (A/P) and dorsal/ventral (D/V) compartment boundaries, such as Dpp and wingless respectively [2]. Although wingless appears to be the main growth-promoting signal emanating from the D/V boundary, a second Notch-induced signal at the D/V boundary also non-autonomously induces wing growth [5]. The wingless paralog Wnt6 is also expressed at the D/V boundary [6]. Therefore, we decided to test whether Wnt6 might constitute this second signal.

*Drosophila* is one of the model systems in which Wnt signaling and function have been most intensively studied. *Drosophila* has seven Wnt genes. Of these, the best understood is wingless, the founding member of the class. Wingless has a myriad of functions during development. One function is to pattern epidermal cells to form repetitive patterns of naked cuticle and denticle belts, small tooth-like structures used for traction during larval crawling [7,8]. Wingless is also necessary for development of all imaginal discs – the tissues resident in the larva which will give rise to adult tissues during metamorphosis. For instance, in the wing disc, early expression is responsible for specifying the wing primordium whereas later expression sets up the D/V axis of the wing [8,9]. The remaining Wnts are comparatively less well studied; nonetheless, some functions are known for four of the remaining Wnts: Wnt3 is involved in axon guidance as well as salivary gland migration, Wnt2 regulates salivary gland migration, tracheal development and testis morphogenesis, Wnt8 is part of the Toll/Dorsal signaling network which both specifies the D/V axis of the embryo and participates in the immune response, whereas Wnt4 is critical for the regulation of cell motility during ovarian morphogenesis (reviewed in [10]). The functions of Wnt6 and Wnt10, however, are not known.

Wnt6 function has been studied in Xenopus, where it was found to be expressed in tissues close to and inside the developing heart, where it regulates heart organogenesis [11]. Since, to our knowledge, Wnt6 mutant flies have not been reported, we generated Wnt6 knockout flies. We find that Wnt6 knockout flies, however, do not have growth defects in the wing. Instead, they completely lack maxillary palps. Together with antenna, maxillary palps are one of two olfactory epithelia in *Drosophila*[12]. Recent studies suggest maxillary palps might also be involved in taste enhancement [13]. The function of maxillary palps as an olfactory organ is well conserved throughout insects. For instance, mosquitos use maxillary palps to smell CO_2_, which is used for host seeking behavior [14]. Hence, since Wnt6 knockout flies lack maxillary palps, they might constitute a useful tool for studying olfaction and behavior [13,15].

## Results

### Wnt6 knockout flies lack maxillary palps

**Figure 1:**
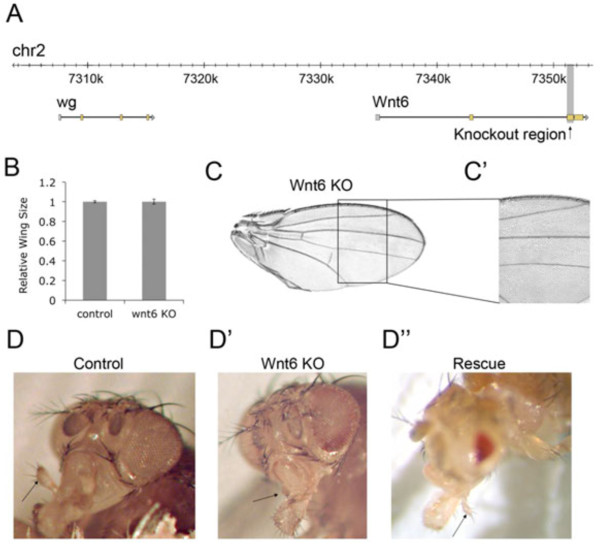
**Wnt6 is required for maxillary palp but not wing formation. (A)** Wnt6 genomic locus showing the knocked-out region (grey area). **(B)** Wings from Wnt6^KO^ adult males are not altered in size compared to controls. **(C-C’)** Wnt6^KO^ wings display grossly normal patterning **(C)** and no obvious planar-cell polarity phenotype **(C’)**. **(D-D”)** Wnt6^KO^ flies **(D’)** lack maxillary palps (positions indicated with arrows). Pan-neuronal re-expression of Wnt6 from a UAS transgene using elav-GAL4 in Wnt6^KO^ animals restores maxillary palp formation **(D”)**. The additional ectopic expression in brain and eye discs causes reduced overall head size and rough eyes. Wnt6^KO^, Wnt6 knockout.

To study the developmental function of Wnt6 in *Drosophila*, we generated Wnt6 knockout (Wnt6^KO^) flies by targeted homologous recombination, entirely removing the third exon and, hence, a substantial portion of the coding sequence (Figure 1A and Additional file 1: Figure S1A), yielding flies with no detectable Wnt6 transcript [see Additional file 1: Figure S1B-D’]. Wnt6^KO^ flies are viable and fertile and have no obvious defects in wing size (Figure 1B-C). The wing margin, specified via wingless signaling, appears intact, and there is no obvious Planar Cell Polarity (PCP) phenotype (Figure 1C’). In sum, Wnt6 does not seem to regulate wing size or PCP signaling. Wnt6^KO^ animals, however, completely lack maxillary palps, structures used by flies for olfaction Figure 1D-D’ and Additional file 2: Figure S2) [13,15]. We asked whether reintroducing Wnt6 expression via a UAS transgene would rescue maxillary palp formation in the Wnt6^KO^. To our knowledge, however, no GAL4 drivers exist with maxillary palp-specific expression. We, therefore, expressed UAS-Wnt6 ubiquitously in the nervous system of Wnt6^KO^ flies, including the maxillary palp anlage, using elav-GAL4. This leads to expression both in the maxillary palp territory as well as elsewhere. Although this caused morphological defects, such as small heads and glazed eyes, this rescued maxillary palp formation (Figure 1D”), confirming that the defect is specific for Wnt6 loss of function. (The Wnt6 overexpression phenotypes observed in the head are similar to those seen for wingless gain-of-function in the eye which disrupts photoreceptor and ommatidial bristle formation [16].)

### Wnt6 can activate canonical Wnt signaling and is required for proper positioning of wing margin chemosensory bristles

**Figure 2:**
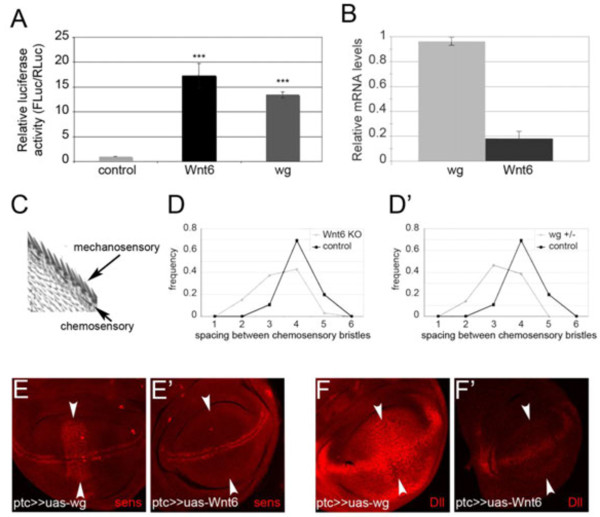
**Wnt6 can activate canonical wingless signaling. (A)** Wnt6 can activate canonical wingless signaling when overexpressed in S2 cells. Activity of a Wnt-responsive LEF_7_-luciferase reporter is shown for cells co-transfected with a control plasmid or plasmids expressing Wnt6 or wingless. n = 3 Error bars: std dev. ***t-test ≤0.001 relative to control. **(B)** Expression levels of Wnt6 are low compared to wingless in wing discs. mRNA levels quantified by quantitative RT-PCR on w^1118^ wing discs. **(C)** Image of an anterior wing margin indicating positions of chemosensory and mechanosensory bristles. The spacing between two chemosensory bristles is on average four times the spacing between mechanosensory bristles. **(D-D’)** Spacing of chemosensory bristles is aberrant in adults wings of both Wnt6^KO^ **(D)** and wingless hypomorphic (wg^1-8/+^) animals **(D’)**. Histogram of chemosensory bristle spacing, measured using the spacing between two mechanosensory bristles as the unit length. **(E-F’)** Overexpression of wingless **(E and F)** but not Wnt6 **(E’ and F’)** in the patched domain with patched-GAL4 (perpendicular to the dorsal/ventral margin, arrowheads) causes upregulation of the wingless target genes, senseless (sens) and Distalless (DII). std dev, standard deviation; Wnt6^KO^, Wnt6 knockout.

Since Wnt6^KO^ flies do not have PCP phenotypes, we tested whether Wnt6 can activate canonical Wnt signaling. Indeed, in S2 cells, Wnt6 was able to induce expression of a Wnt-responsive LEF_7_-luciferase reporter [17] in a manner similar to wingless (Figure 2A). Given that Wnt6 can activate canonical wingless signaling, we were surprised that loss of Wnt6 did not lead to obvious wing margin defects. One possible explanation could be that Wnt6 is expressed at lower levels than wingless in the wing disc. Indeed, quantitative RT-PCR on RNA extracted from wing discs confirmed this to be the case (Figure 2B). A second possible explanation that is not mutually exclusive with the first is that Wnt6 might be less effective at activating wingless signaling in the wing disc compared to wingless. Indeed, expression of wingless with patched-GAL4 in a domain along the A/P boundary, perpendicular to its normal expression domain, caused ectopic expression of canonical target genes, such as senseless (sens) and distalless (Dll) (arrowheads in Figure 2E and 2F). In contrast, expression of Wnt6 in this same domain had no clear effect on sens or Dll expression (Figure 2E’ and 2F’). In sum, in the wing disc Wnt6 appears to induce Wnt signaling significantly less strongly than wingless itself. We, therefore, asked whether Wnt6^KO^ flies might have a mild wingless loss-of-function phenotype in the wing that had escaped our initial inspection. Wingless signaling specifies the mechanosensory and chemosensory bristles along the anterior wing margin [18], with the spacing between chemosensory bristles normally being four times larger than the spacing between stout mechanosensory bristles (Figure 2C). We noticed that the spacing of chemosensory bristles in Wnt6^KO^ wings tends to be less precisely specified, with a reduced average distance and a broader spacing distribution, compared to controls (Figure 2D). The same was observed for flies with reduced wingless signaling due to heterozygosity for a wingless amorphic allele (wg^1-8/+^) (Figure 2D’).

### Wnt6 is expressed in the maxillary palp anlage

**Figure 3:**
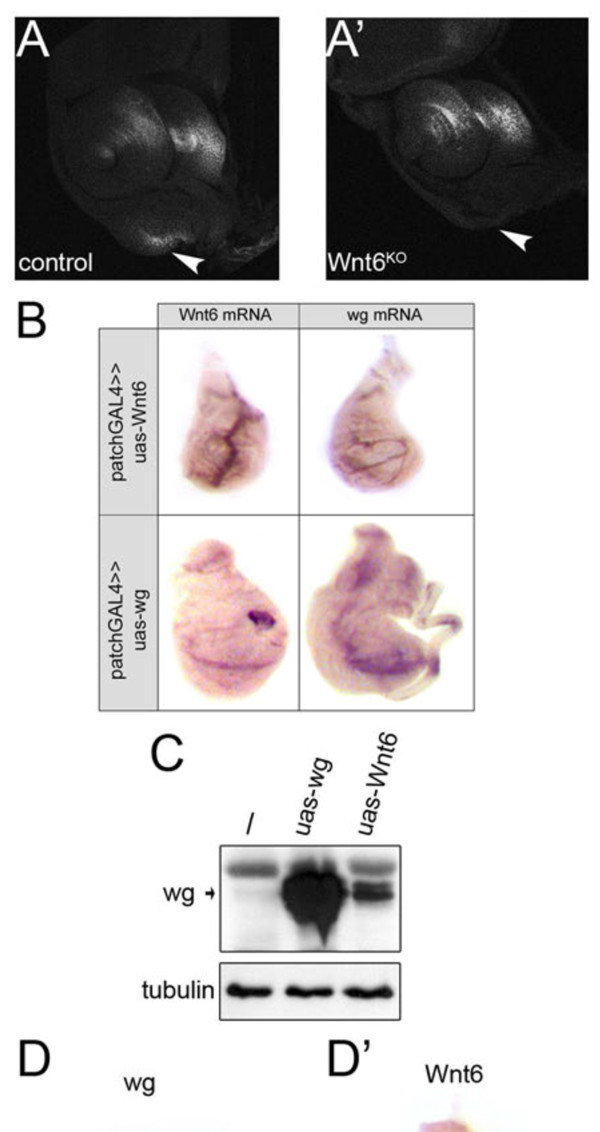
**Wnt6 is expressed in the maxillary palp anlage, and not wingless. (A-A’)** Staining of control eye-antenna discs **(A)** with anti-wingless antibody (clone 4D4) gives a signal in the maxillary palp anlage (arrowhead) which is not visible in Wnt6^KO^ discs **(A’)**. **(B)** Wingless and Wnt6 do not induce expression of each other, detected by mRNA *in situ* hybridization. Expression of Wnt6 in the patched domain along the A/P boundary, perpendicular to the endogenous wingless and Wnt6 expression domains, does not ectopically induce wingless, and vice-versa. **(C)** Anti-wingless monoclonal antibody clone 4D4 cross-reacts with Wnt6 on immuno-blots. Lysates from control S2 cells or S2 cells transfected to express wingless or Wnt6 were probed with anti-wg antibody. The upper band is a non-specific band also present in untransfected S2 cells. **(D-D’)***In situ* hybridization detecting wingless mRNA **(D)** and Wnt6 mRNA **(D’)** in eye-antenna discs reveals Wnt6 expression but not wingless expression in the maxillary palp anlage (arrow). Wnt6^KO^, Wnt6 knockout.

Previous work has shown that Wnt signaling in the maxillary palp anlage is required for growth of the maxillary palp [19]. Indeed, as previously reported [19], anti-wingless antibody 4D4 detects a protein in the maxillary palp territory of the eye-antenna disc of control animals (arrowhead Figure 3A), suggesting wingless might be the ligand activating the pathway. This antibody signal, however, is gone in Wnt6^KO^ animals (arrowhead Figure 3A’). One possible explanation is that induction or maintenance of wingless expression requires Wnt6. However, we could not find any cross-talk between Wnt6 and wingless expression; expression of Wnt6 using patched-GAL4 did not induce wingless expression and vice-versa (Figure 3B). An alternate explanation is that the anti-wingless 4D4 antibody might cross-react with Wnt6 protein. Indeed, expression of Wnt6 in S2 cells yielded a clear signal with anti-wingless 4D4 antibody on an immunoblot (Figure 3C). Consistent with this explanation, by *in situ* hybridization we could detect Wnt6 expression and not wingless expression in the maxillary palp anlage (arrows Figures 3D-D’), suggesting that the antibody staining might be detecting Wnt6 and not wingless. In sum it appears that canonical Wnt signaling in response to Wnt6 ligand is responsible for maxillary palp formation, although we cannot exclude the possibility that Wnt6 is promoting maxillary palp growth via a non-canonical pathway.

### Evolutionary loss of Wnt6 correlates with loss of maxillary palps

**Figure 4:**
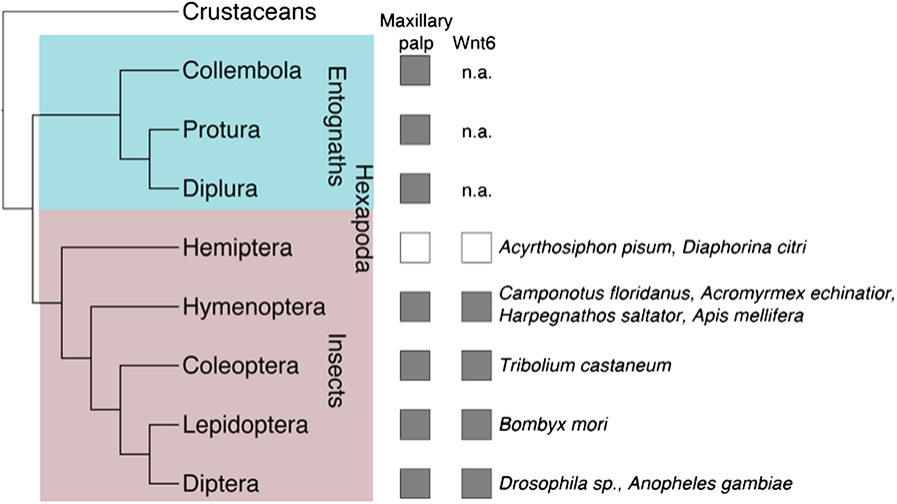
**Correlation between the presence/absence of Wnt6 and maxillary palps in insects.** Simplified phylogenetic tree of hexapoda, based on [2]. The presence or absence of maxillary palps and Wnt6 are indicated on the right. The insect species with whole genome information are listed. n.a. – no genomic information available. The position of hemiptera nested within groups with maxillary palps indicate that the absence of maxillary palps in this group is due to evolutionary loss.

Since Wnt6^KO^ flies lack maxillary palps, we asked whether the presence or absence of maxillary palps across hexapods correlates with the presence or absence of the Wnt6 gene. Hexapods include ectognaths (exposed jaws; Insecta sensu stricto) and entognaths (enclosed jaws). The minute, wingless entognaths have three orders, Protura, Diplura and Collembola, and represent the sister group to insects [20,21]. Maxillary palps are present in most insects and also in entognaths, indicating that the structure was present in their last common ancestor. Notably among the insects, aphids and other hemipterans lack maxillary palps due to evolutionary loss (Figure 4). A survey of 10 insect species for which complete genomic information is available revealed that all of these species have a Wnt6 gene except the aphid, *Acyrthosiphon pisum*[22], and the jumping plant lice (Psylloidea) *Diaphorina citri* (Figure 4), both belonging to Hemiptera. Wg/Wnt1, the closest paralog to Wnt6 [23], and other Wnts are present in *A. pisum*[22] and could also be identified it in the *D. citri* genome [24]. This finding suggests that loss of Wnt6 may have led to the loss of maxillary palps also on the evolutionary timescale during hemipteran evolution.

## Discussion

Wnt6 appears to have a specific role during *Drosophila* development, promoting maxillary palp formation. This is surprising, given that Wnt6 is quite ancient and present in most bilaterians [25,26]. One possible interpretation is that the Wnt6 function might be redundant in most parts of the animal, perhaps due to overlapping expression with wingless, whereas Wnt6 expression in the maxillary palp might have been acquired in insects in a non-redundant fashion. This specific function in promoting maxillary palp formation might serve as a useful tool for studying the contribution of maxillary palps to olfaction and behavior. The maxillary palp contribution is currently assayed by surgical removal of the palps, whereas this could now also be accomplished genetically [27].

As previously noted [28], the Wnt6 gene is located directly adjacent to the wingless gene, raising the possibility that it arose as a genomic duplication of wingless. Accordingly, Wnt6 expression overlaps with that of wingless in numerous places [6]. One possible reason for the overlapping expression patterns could be that Wnt6 expression is induced by wingless signaling; however, our data suggest this is not the case (Figure 3B). Instead, it is likely that they either share enhancer elements or that regulatory elements were also duplicated alongside the open reading frame. Since wingless and Wnt6 have similar expression patterns and presumably transcriptional regulation, and since the anti-wingless monoclonal antibody 4D4, the most widely-used in the field to detect wingless, appears to cross-react with Wnt6, some caution might be warranted in interpreting results with this antibody.

Given that Wnt6 is able to induce canonical wingless signaling in S2 cells (Figure 2A), we were surprised that Wnt6 is quite poor at inducing wingless signaling in the wing disc (Figure 2E-F’). Consistent with this observation, expression of UAS-Wnt6 with various GAL4 drivers such as patched^ts^-GAL4 (with GAL80^ts^) and nubbin-GAL4 cause pupal lethality; however, this does not yield obvious morphological defects in the resulting wings (not shown), suggesting that the lethality is likely due to expression in other parts of the body. In contrast, Wnt6 expression in the central nervous system, including the maxillary palp, induces obvious significant morphological effects. One possible explanation could be that a component required for Wnt6 signaling might be expressed at higher levels in the nervous system compared to wing discs.

The specific absence of Wnt6 from the aphid *A. pisum* and the plant lice *D. citri,* both belonging to the order Hemiptera, a group that has lost maxillary palps, suggests that Wnt6-loss could have been the underlying genetic alteration leading to this morphological change. In hemipterans, the mouthparts are modified to form a tube-like structure for piercing. The tube, formed by the labrum and labium, comprises piercing-sucking structures formed by the modified mandible and the maxilla [29]. The evolutionary loss of the maxillary palps was one of many structural modifications leading to the specialized hemipteran mouthparts. The loss of the maxillary palps could have compromised the sense of smell in hemipteran ancestors, but this may have been compensated by the elaboration of sensory structures on the labium [29]. The specific phenotype of the Wnt6 knock-out in *Drosophila* contrasts with the pleiotropic effects of other secreted signaling molecules including wingless. This means that the deletion of the gene, rather than tinkering with its regulatory regions, could have resulted in a subtle morphological change, the loss of the maxillary palp, contributing to the morphological evolution of the beak-like hemipteran mouthparts.

## Conclusions

Although Wnt6 expression overlaps substantially with that of wingless, it appears to play a critical role in maxillary palp growth, but not wing growth. Phylogenetic analysis suggests that loss of Wnt6 also correlates with loss of maxillary palps on an evolutionary timescale.

## Methods

### Oligos

All oligo sequences are listed in Additional file 3.

### Generation of Wnt6^KO^ and UAS-Wnt6 flies

Wnt6^KO^ flies were generated by homologous recombination-based targeting using the 'ends-out’ strategy as previously described [30]. Based on this strategy, 4 kb upstream and downstream flanks were amplified by PCR using the oligos described in Additional file 3, sequenced, and cloned into the pRK1 vector [30]. Knockout flies were then back-crossed to the w^1118^ reference strain for five generations before studying. To generate UAS-Wnt6 flies, the Wnt6 ORF was amplified by PCR using the oligos listed in Additional file 3, and cloned into the EcoRI\NotI sites of pUAST [31].

### Luciferase reporter assays

Luciferase reporter assays were based on the Promega pGL3 reporter system with a Wnt-responsive LEF_7_ firefly luciferase reporter and a renilla normalization control as previously described [17]. Wingless and Wnt6 expression were achieved by co-transfecting pac-wg or pUAST-Wnt6 + pMT-GAL4/VP16, respectively [17].

### Cell culture

S2 cells were grown in Express Five Serum Free Medium (Life Technologies, Carlsbad, California, USA) and transfected with Effectene (Qiagen, Venlo, Netherlands).

### Antibodies

Antibodies used were mouse anti-wingless (4D4 Developmental Studies Hybridoma Bank); rat anti-Dll (Sean Carroll lab, R.M Bock Laboratories, 1525 Linden Drive, Madison, WI 53706) and guinea pig anti-sens [32].

### *In situ* hybridization

*In situ* hybridizations were carried out as previously described [33,34], using digoxigenin-labeled RNA probes, an AP-conjugated digoxigenin antibody (Roche, Basel, Switzerland).

### Bioinformatics

The *D. citri* genome (Diaci1.1, 12× coverage) and transcriptome assemblies (Diaci_transcriptome_0.9) were downloaded from the International Asian Citrus Psyllid Genome Consortium website [24] and searched with TBLASTN with arthropod Wnt6 query sequences. The five top hits were re-blasted to the Uniprot and GenBank databases. *D. citri* Wnt1 is on the genomic scaffold scaffold5281.1|size8182|ref0095796|ref0108241.

## Abbreviations

A/P: anterior/posterior; D/V: dorsal/ventral; DII: distalless; PCP: planar cell polarity; sens: senseless; wg: wingless.

## Competing interests

The authors declare that they have no competing interests.

## Authors’ contributions

ND helped design and performed all wet-lab experiments. GJ performed the evolutionary analysis and helped write the manuscript. AT designed experiments and helped write the manuscript. All authors read and approved the final manuscript.

## Supplementary Material

Additional file 1: Figure S1 Wnt6^KO^ flies have no detectable Wnt6 mRNA. (A) Quantitative PCR on genomic DNA from control and Wnt6^KO^ flies confirming absence of the Wnt6 knockout region, but not absence of two other unrelated genomic regions (mir-278 and sty), in the knockout flies. (B) Quantitative RT-PCR to detect Wnt6 mRNA levels in control and Wnt6 knockout flies reveals no Wnt6 transcript left in the knockouts. (C-D’) *In situ* hybridization to detect Wnt6 mRNA in wing discs (C-C’) and eye-antenna discs (D-D’) of control and Wnt6 knockout animals. Click here for file

Additional file 2: Figure S2 Wnt6^KO^ flies lack maxillary palps. (A-C) Scanning electron micrographs showing that Wnt6^KO^ flies (B), unlike control flies (A), lack maxillary palps (positions indicated with arrows). Click here for file

Additional file 3 Oligo sequences. Click here for file
