# Erratum to: Wnt6 is required for maxillary palp formation in Drosophila

**DOI:** 10.1186/s12915-015-0209-2

**Published:** 2015-11-25

**Authors:** Nikolaos Doumpas, Gáspár Jékely, Aurelio A. Teleman

**Affiliations:** German Cancer Research Center (DKFZ), Heidelberg, Germany; Max Planck Institute for Developmental Biology, Spemannstrasse 35, 72076 Tuebingen, Germany

## Correction

We realized that our 2013 publication describing Wnt6 knockout flies [1] contains a presentation error which does not impact any of the data or conclusions of the manuscript.

Figure 1a, illustrating the genomic region that was knocked-out in the Wnt6 [KO] flies, is not correct. The knockout takes out exon 1, not exon 3. Hence the gray shading in Figure 1a is in the wrong spot, and the corresponding text in the Results section is not correct. The actual knockout allele removes exon 1 of Wnt6, and its translation start site, also leading to complete loss of function. This happened because we originally had two different knockout strategies - one to take out exon 1, which was successful, and one to take out exon 3, which did not yield knockout animals – and this got mixed up when writing the paper. In the Supplemental Materials of the paper we provided oligo sequences describing the generation of the knockout vector, and those are correct.

We believe this does not impact the results or interpretation of the data. Both of the knockout strategies were designed to yield null alleles. We checked by Q-RT-PCR that no Wnt6 transcript is detectable in the knockout flies (Figure S1B-D’) of the paper. The phenotypes described in the paper were rescued with a UAS-Wnt6 transgene, showing they are specific.

Nonetheless, the molecular description of the allele is incorrect, and this can cause confusion when molecularly genotyping the mutant flies, hence we felt it necessary to publish this Correction.
