# Supplementary material for: Wnt6 is required for maxillary palp formation in Drosophila
Source: BMC Biol. 2013 Oct 3;11:104. doi: 10.1186/1741-7007-11-104 (PMC3854539; doi:10.1186/1741-7007-11-104)
Supplement: Additional file 3 — Oligo sequences. [file 1741-7007-11-104-S3.pdf]

Wnt6 is required for maxillary palp formation in Drosophila

Doumpas et al.

Supplemental Information

Oligo sequences

•To amplify Wnt6 upstream flank for the knockout vector:
ctgcagTGGCCGCGCACTCAGCAC
ggcgcgccCGCCGTCGAAAAGTCTCT

•To amplify Wnt6 downstream flank for the knockout vector:
gctagcGATTCGGCTGGTAAGTGG
gcggccgcCGATGCCAGGCAATGTCA

•To amplify Wnt6 ORF to make UAS-Wnt6:
gaattcATGCGTTTGCTCATGGTAAT
GCGGCCGCtcagaggcaggtgttgaccg

•Wnt6 in situ probe by T7 transcription:
TGCGGCTGGAGTGCAAG
taatacgactcactatagggaggCGATCCGGAACTGGTCACA

•wingless in situ probe by T7 transcription:
AGCGGAGATGCGACAGGAG
taatacgactcactatagggaggCAGGCCGTTGGAGCCCACG

•Wnt6 Q-RT-PCR primers:
ATTTGGCGGCTTCAATC
GCGCAGACGACGTGTCT

•wingless Q-RT-PCR primers:
TGCGCGAGAAGATGAATC
CGCATCCAGCAGGTCTTC
