# Supplementary material for: Wnt6 is required for maxillary palp formation in Drosophila
Source: BMC Biol. 2013 Oct 3;11:104. doi: 10.1186/1741-7007-11-104 (PMC3854539; doi:10.1186/1741-7007-11-104)

Doumpas et al.
Supplemental Figure 1

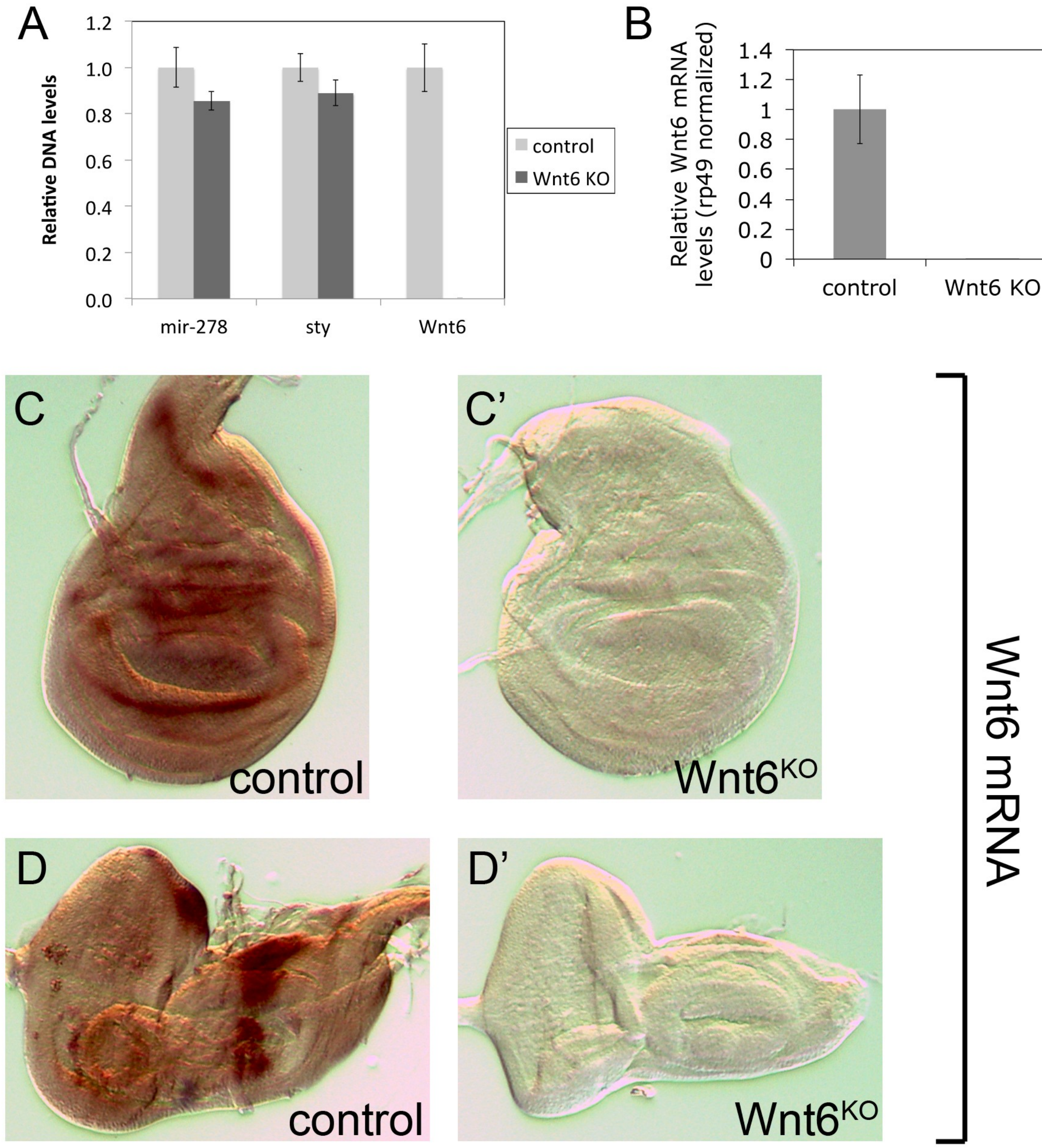

Supplement: Supplementary file 2 [file 1741-7007-11-104-S1.doc]
